# Möglichkeiten und Grenzen der konservativen Therapie der Arthrose: Sportberatung, Trainingstherapie, Orthesen und Knorpeltherapeutika

**DOI:** 10.1007/s00132-021-04100-0

**Published:** 2021-04-09

**Authors:** Stefan Nehrer, Markus Neubauer

**Affiliations:** 1 Zentrum für Regenerative Medizin, Donau-Universität Krems, Dr. Karl-Dorrek-Straße 30, 3500 Krems, Österreich; 2 Universitätsklinik für Orthopädie, Universitätsklinikum Krems, Karl Landsteiner Privatuniversität für Gesundheitswissenschaften, Krems, Österreich

**Keywords:** Altern, Athletik, Orthesen, Konservative Behandlung, Gelenke, Aging, Athletics, Braces, Conservative therapy, Joints

## Abstract

**Hintergrund:**

Arthrose – die Degeneration von Gelenken – ist ein weit verbreitetes Problem durch alle Bevölkerungsschichten, das im zunehmenden Alter vermehrt auftritt und die häufigste Ursache für mobilitätseinschränkende Schmerzen am Bewegungsapparat ist. Etwa 70–80 % der über 70-Jährigen zeigen Zeichen einer Gelenksdegeneration. Insgesamt sind bis zu 25 % der Gesamtbevölkerung davon betroffen, aufgrund der generellen Alterung der Bevölkerung mit steigender Tendenz. Die Inzidenz der Arthrose steigt aber schon ab dem 40 Lebensjahr, wobei besonders posttraumatische und sekundäre Arthroseformen zum Tragen kommen.

**Anspruch:**

Der Wunsch nach hoher Mobilität und Sport zieht sich als Phänomen ebenfalls durch alle Altersgruppe. Dies ist mit hohen Gelenkbelastungen verbunden und stellt damit eine große Herausforderung an vor allem früh degenerativ veränderte Gelenksstrukturen dar. In diesem Zusammenhang ist der orthopädisch tätige Arzt gefordert, die Belastbarkeit von geschädigten Gelenken abzuschätzen und so früh wie möglich präventive Schritte sowie gegebenenfalls konservative Therapien einzuleiten, um die Progression der Arthrose zu verhindern und damit den eventuell notwendigen Gelenkersatz möglichst weit nach hinten zu schieben.

## Abstract (en)

**Background:**

Osteoarthritis (OA)—the progressive degeneration of synovial joints—is a worldwide problem that affects society, is associated with age and is the dominant reason for pain and immobility of the locomotor system. In the population, 70% of people over the age of 70 show some signs of OA. Overall, up to 25% of the total population is affected, due to the general aging of the population itself, and this is an increasing tendency. However, in people over the age of 40 the incidence of OA is already rising due to posttraumatic or secondary forms, like dysplasia, malalignment and other background factors.

**Claim:**

There is a high need for mobility and sports in all generations, so the demands on the joints are high, especially if early degenerative changes are already present. Orthopaedic physicians are challenged with the limited load capacity, pain and the progressive joint problems, on the one hand to get the patient back to full mobility, and, on the other, to prevent early joint replacement. Early diagnosis and preventive measurements, as well as conservative treatments—from medication to braces—are necessary to achieve symptom-free movement and joint preservation to avoid joint replacement.

Arthrose – die Degeneration von Gelenken – ist ein weit verbreitetes Problem durch alle Bevölkerungsschichten, das im zunehmenden Alter vermehrt auftritt und die häufigste Ursache von mobilitätseinschränkenden Schmerzen am Bewegungsapparat ist. 70–80 % der über 70-Jährigen zeigen Zeichen einer Gelenksdegeneration. Insgesamt sind bis zu 25 % der Gesamtbevölkerung davon betroffen, und aufgrund der generellen Alterung der Bevölkerung mit steigender Tendenz. Die Inzidenz der Arthrose steigt aber schon ab dem 40. Lebensjahr, wobei besonders posttraumatische und sekundäre Arthroseformen zum Tragen kommen.

## Einleitung

Der Wunsch nach hoher Mobilität und Sport zieht sich als Phänomen durch alle Altersgruppen. Diese Aktivitäten sind mit hohen Gelenkbelastungen verbunden und stellen damit eine große Herausforderung an degenerativ veränderte Gelenksstrukturen dar. In diesem Zusammenhang ist der orthopädisch tätige Arzt gefordert, die Belastbarkeit von geschädigten Gelenken abzuschätzen, so früh wie möglich präventive Schritte einzuleiten und gegebenenfalls konservative Therapien einzuleiten, um die Progression der Arthrose zu verhindern und damit den eventuell notwendigen Gelenkersatz möglichst weit nach hinten zu schieben.

## Grundlagen: Gelenkknorpel und Arthrose

Der Gelenkknorpel besteht beim Erwachsenen zu etwa 5 % aus Zellen, den Chondrozyten oder Knorpelzellen, zu 20–40 % aus Kollagen- und Glykosaminoglykanen als Matrix und bis zu 60 % aus Wasser. Er besitzt keine Gefäß- oder Nervenversorgung. Die Zellernährung erfolgt durch Diffusion. Der Verbund aus Matrix und Wasser bildet die Grundlage für die biomechanischen und viskoelastischen Eigenschaften des Knorpels. Die eingebundene Flüssigkeit im Gelenkknorpel führt bei Belastung zur Dämpfung und die Abpressung derselben an der Knorpeloberfläche trägt wesentlich zur Gleitfähigkeit und den optimalen Schmiereigenschaften von Knorpel bei. Die Ultrastruktur des Kollagengerüstes und der darin gebundenen Glykosaminoglykane, die für die Wasserbindung verantwortlich sind, stellen das biomechanische Konzept des Knorpels dar. Verletzungen dieser Ultrastruktur, durch kritische traumatische Impacts, können zu mikrotraumatischen Veränderungen mit Rupturen im Kollagengerüst führen. Nachfolgend kommt es einerseits zur Ausschwemmung der Glykosaminoglykane, andererseits können Traumata zu einem Ausbruch von Knorpelschuppen oder osteochondralen Fragmenten führen. Der isoliert traumatische Knorpeldefekt als Ursprung von Gelenksdegeneration ist häufig, aber durchaus nicht die einzige Ursache der Arthroseentstehung, die einen sehr vielfältigen, auch organischen Hintergrund haben kann, und mit einer verminderten Belastbarkeit in osteochondralen Strukturen einhergeht.

Ein Zusammenspiel von intrinsischen und extrinsischen Faktoren ist ursächlich für die Arthroseentstehung

In Summe kann ein komplexes Zusammenspiel von intrinsischen und extrinsischen Faktoren als ursächlich für die Arthroseentstehung angesehen werden. Dabei steht das Dreieck aus Alter – Degeneration – Inflammation im Zentrum [1].

Alter ist dabei mit morphologisch abnehmender Knorpeldicke, weniger Proteoglykanen, einer geringeren Kollagendichte und Dedifferenzierungen vergesellschaftet [2]. Im Weiteren kommt es zu einem proteolytischen Matrix-Breakdown an der Oberfläche des Knorpels mit beginnender Fibrillation, Aufschwellung des Knorpels, dann Ausschwemmen der Glykosaminoglykane und Zerreißen der Kollagenstrukturen, bis hin zum völligen Knorpelverlust mit Abrieb der Gleitoberfläche. Das Altern auf zellulärer Ebene ist ein geplanter Prozess der sehr komplex ist und mit dem Auftreten von charakteristischen, seneszenten Zellmerkmalen verbunden ist. Knorpelzellen ändern ihren Status hin zu einem „senescence-associated secretroy phenotype“, unter anderem über die Ausschüttung der Mediatoren IL‑1, IL‑6 oder Metalloproteinase‑3. Das gezielte Eliminieren seneszenter Zellen oder Merkmalen mit „Senolytics“ rückt daher bei degenerativen Erkrankungen als vielversprechende therapeutische Strategie in den Fokus [3].

Neben der mechanischen Betrachtungsweise der Gelenksdegeneration setzt parallel auch ein biologischer Prozess der Gelenkdestruktion und des Knorpelabbaus ein. Soul et al. haben in einer rezenten Arbeit zur biologischen Grundlage von Arthrose im Wesentlichen 2 unterschiedliche Osteoarthrosegruppen beschreiben können [4]. Die wesentlichen Unterschiede dabei waren die zur Krankheitsinduktion und -progression beitragenden Prozesse (Wnt- und TGFβ-Signalwege), zusammen mit einer veränderten Antwort der angeborenen Immunität und Komplementaktivierung.

Grundsätzlich bewirkt der Knorpelabrieb einen synovialen Reizzustand, der über Interleukin‑1 und TNF‑α eine Entzündungsreaktion in Gang setzt. Durch diese Reaktionen werden auch Metalloproteasen (Kollagenasen) freigesetzt, die als zersetzende Enzyme das Kollagengerüst zusätzlich angreifen und rasch zerstören. Dieser entzündliche Vorgang ist auch jener, der für das schmerzhafte Erscheinungsbild der Arthrose hauptverantwortlich ist, das mit Ergussbildung, Bewegungseinschränkung und allgemein eingeschränkter Funktion einhergeht [5]. Die Arthrose kann im Wesentlichen alle Gelenke betreffen, hauptsächlich aber steht die Arthrose im Kniegelenk im Vordergrund sowie die im Sprunggelenk, gefolgt von der Hüfte und dem Schulterbereich. Die Entstehung solcher Gelenkschäden wird natürlich durch Verletzungen, die auch im Sport häufig sind, forciert [6]. Im Knie sind dies meist Meniskus‑, Kreuzband- und Knorpelverletzungen, im oberen Sprunggelenk Bandinstabilitäten und Knorpelkontusionen sowie Kapselverletzungen an der Hüfte, wo vor allem Labrumläsionen und Hüftimpingements Auslöser eines arthrotischen Prozesses sein können [7, 8]. Auch dysplastische Veränderungen, wie sie im Bereich des Hüftgelenkes vorkommen, haben vergleichbare Effekte. Die chronischen Überlastungssituationen in Gelenken, insbesondere im Kniegelenk, können besonders durch O‑ und X‑Bein-Stellung akzentuiert werden.

Das osteoarthrotische Gelenk ist vor diesem Hintergrund wie ein komplexes Organ zu betrachten, bei dem äthiopathologische Faktoren übergreifend mechanisch, biologisch und genetisch zu Krankheitsinduktion und Progression beitragen. Dieses degenerativ veränderte Organ hat in Konsequenz auch systemische Auswirkungen auf den Gesamtorganismus, dabei spielen vor allem die zirkulierenden Inflammationsmediatoren eine Rolle [9].

**Figure 1:**
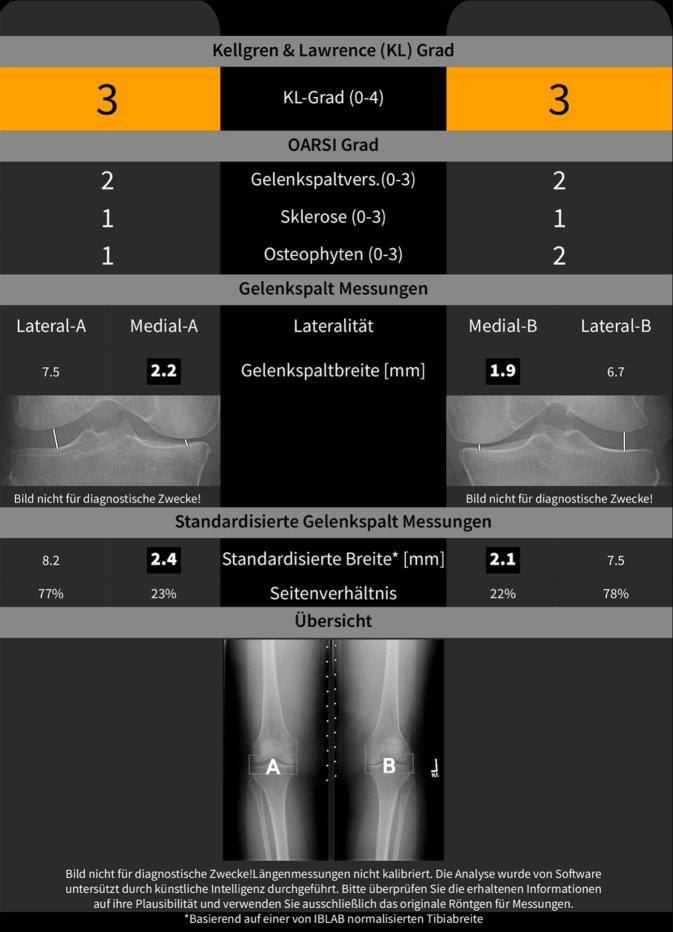

Radiologisch äußert sich die fortschreitende Arthrose durch eine Verschmälerung des Gelenksspaltes, eine Zunahme der subchondralen Sklerose mit progressiver Zystenbildung in den gelenknahen Strukturen, Kalzifizierung und Osteophytenbildung sowie einer zunehmenden Achsabweichung aufgrund der damit verbundenen Bandinstabilitäten und Knochendeformationen. Die Gradierung der Arthrose erfolgt in klassischer Weise nach dem Kellgren-Lawrence-Score, der subjektiv semiquantitativ vier Kriterien (Gelenkspalt, Sklerosierung, Verformung und Osteophyten) erfasst, damit aber eine geringe inter- und intraindividuelle Reproduzierbarkeit aufweist. Die Anwendung von digitalen Analyseverfahren in der muskuloskelettalen Bildgebung ermöglicht es, vollautomatisch mithilfe neu entwickelter Algorithmen konventionelle Röntgenbilder auf Frühzeichen einer Arthrose zu untersuchen. Dadurch lässt sich die hohe Befundvariabilität des Kellgren-Lawrence-Scores und damit auch die Datenqualität in Arthrosestudien deutlich verbessern [10]. Die Analyse des gesamten Datensatzes eines digitalen Röntgenbildes lässt auch Analysen der ossären Mikrostruktur zu, wobei Aufbau und Zusammensetzung des subchondralen Knochens im Vordergrund stehen. Durch „deep learning“-Algorithmen der künstlichen Intelligenz können Frühzeichen der Arthrose detektiert werden, wodurch Risikoprofile und Prognosewerte für die Arthroseentstehung generiert werden und somit therapeutische bzw. präventive Maßnahmen rechtzeitig begonnen werden können ([11]; Abb. 1).

## Muskelatrophie und Arthrose

Die Arthrose ist auf muskulärer Ebene mit sekundären Muskelpathologien assoziiert, nämlich der strukturellen Muskelatrophie und der funktionellen Muskelschwäche [12–14]. Bewegungseinschränkung als offensichtliche Ursache der Muskelatrophie stellt dabei im Rahmen der Arthroseentwicklung nur einen Teil der vielfältigen Ursachen dar [14]. Die arthrogene Muskelinhibition ist ein anderer zentraler Faktor in der graduellen Abnahme von Muskelkraft und -masse bei Arthrose. Als Synonym wird in der Literatur der Begriff „Reflexarthropathie“ verwendet. Dies bezeichnet eine spezifische Reaktion des Nervensystems, die sogenannte arthrogene Muskelinhibition [15]. Aufgrund von Signalen aus anormalen nozizeptiven Gelenkafferenzen kommt es zu einer Neurotransmitterfreisetzung, die eine Aktivitätshemmung der alpha-Motoneurone bewirkt und somit eine sekundäre Muskelatrophie durch Muskelminderaktivität verursacht [15].

Muskuläre Dysbalancen sind Folge der Muskelschwäche und führen zu einer Destabilisierung des schon vorgeschädigten Gelenks. Aus der unpräzisen, unkontrollierten Bewegung resultiert eine Gelenksinstabilität mit unkoordinierten Knorpelbelastungen, und damit eine raschere Progredienz der Knorpeldegeneration. Assoziierte Schmerzen hindern Arthrosepatienten häufig, einen erneuten Muskelaufbau zu forcieren. Der resultierende Immobilisationszustand führt zu strukturellen Muskelveränderungen mit funktionellen Einbußen, unabhängig von der Dauer der Immobilisation. Es konnten in diesem Zusammenhang eine Reduktion von Muskelfasern, eine Verminderung der Muskelmasse und biomechanische Veränderungen aufgezeigt werden [16–18].

In den letzten Jahren wurden mehrere Studien publiziert, die nachweisen, dass Muskelatrophie auch bereits vor Auftreten der ersten Arthrosesymptome besteht [19–24]. Als multifaktorielle Erkrankung führt die Sarkopenie zu einem generalisierten Muskelabbau mit nachweisbarer Kraftreduktion [25]. Hauptsächlich sind ältere Menschen von dieser Sarkopenie betroffen, es wird aber auch bei jüngeren Erwachsenen beobachtet [26–28]. Wie bei Muskelatrophie durch Immobilisation, ist hier das einzige Therapiekonzept derzeit das regelmäßige Krafttraining. Es sollte dabei ein isokinetisches Muskeltraining durchgeführt werden, bei dem Agonisten und Antagonisten gleichzeitig trainiert werden, bei nur geringem Risiko einer Gelenküberlastung. Das Training muss entsprechend dem Ausmaß der Muskelveränderungen angepasst werden [29], da Personen mit Muskelschwäche und resultierend schlechterer Gelenksstabilisierung ein deutlich erhöhtes Risiko haben, eine fortschreitende degenerative Gelenksveränderung zu entwickeln.

## Trainingstherapie

Die Rationale zur Beurteilung einer „physiologischen“ sportlichen Belastung basiert auf dem Verständnis der inneren Biomechanik der Knorpelstruktur, dabei erscheinen zyklische Belastungen mit Be- und Entlastungsphasen notwendig, um die Knorpelzelle zu ernähren. Wie oben beschrieben, ist die Knorpelzelle abhängig von Diffusion, die durch den Pumpmechanismus der Belastung aufrechterhalten wird. Da sich die viskoelastischen Fähigkeiten von Knorpel erschöpfen, sind Erholungsphasen notwendig. Demgegenüber stehen lang statische Belastungen – wie langes Stehen oder langes Sitzen – die für das Gelenk und die Knorpelstruktur problematisch sein können, da es zum Auspressen der Flüssigkeit und zum Erschöpfen des Schmier- und Dämpfungsmechanismus des Knorpels kommt. Daneben sind kurze Stoßbelastungen, die die Bruchlasten von Knorpel überschreiten immer problematisch. Diese kommen meist im Rahmen von Traumata vor. In jedem Fall erscheint es wichtig, Reizzustände nach (Mikro)Traumata oder chronischer Überlastung zu vermeiden, da die vergesellschaftete Inflammation zur progredienten Gelenkdestruktion beiträgt.

Tierexperimentelle Studien zum Einfluss von Bewegung auf Arthrose konnten zeigen, dass moderate Laufbelastungen bei Hunden den Glukosamingehalt und die Knorpeldicke verbessern konnten. Wurden jedoch die Laufinhalte der Tiere deutlich erhöht – vor allem mit höherfrequenten Belastungen mit längeren Umfängen – konnte eine eindeutige Abnahme der Knorpeldicke und des Glukosamingehaltes festgestellt werden. Niedrigfrequentes, nichtintensives Laufen führte zwar zur Glukosaminabnahme und zu geringer Ausdünnung des Knorpels, parallel jedoch zu einer Verdünnung des subchondralen Knochens ohne Arthrose. Interessant scheint, dass Ausdauertraining mit zyklischer Belastung, eine potenziell positive Adaptierung des Gelenkknorpels zu erlauben scheint und so zu einer Adaptation der Belastbarkeit von Knorpel führen kann. Interessant war auch, dass die Regeneration von Gelenksstrukturen – insbesondere des Knorpels nach länger dauernden Ruhigstellungen – durch übertriebene Laufbelastungen negativ beeinflusst wurden, was den Hinweis gibt, nach längeren Sportpausen einen sorgsamen Wiederaufbau der Belastung auch in diesem Zusammenhang durchzuführen [30].

Bei Sportausübung vom Menschen ergibt sich ein ähnliches Bild in den Studien. Ein erhöhtes Arthroserisiko wurde bei Spitzenläufern festgestellt, wo vor allem hochintensive Langzeitbelastungen mit hohen Schrittfrequenzen und hohem Tempo langfristig das Arthroserisiko steigern. Interessant erscheint auch die Beobachtung, dass Spitzenathleten, obwohl sie teilweise doch vermehrt Arthrosezeichen haben, in der klinischen Symptomatik deutlich besser abschneiden, als Patienten mit ähnlichen radiologischen Arthrosezeichen, die nicht Sport betrieben haben. Für moderates und mittelmäßig intensives Training konnten verschiedene Autoren feststellen, dass kein erhöhtes Arthroserisiko vorliegt und sogar eine bessere Gelenkfunktion zu erwarten ist [31]. Die Umfänge die hier angegeben werden, liegen ungefähr bei 5 h oder 40 km/Woche, wo auch nach langjährigen (5–9 Jahren) Beobachtungen keine vermehrte Arthrose festgestellt werden konnte. In einem Konsensus-Paper von Bosomworth (2009) wurde auch festgehalten, dass moderate Sportausübung, insbesondere Laufen, primär kein Arthrosefaktor ist [32]. Spitzensportler und Eliteläufer haben zwar eine erhöhte Arthrosetendenz zu erwarten, entwickeln dabei aber meist weniger Beschwerden und Einschränkungen. Außerdem sind Sportarten mit abrupten Krafteinwirkungen, hohen Scherkräften und Gegnereinwirkungen, immer mit erhöhtem Arthroserisiko, nicht zuletzt aufgrund der posttraumatischen Veränderungen, verbunden. In jeder Diskussion über Artroseentstehung muss jedoch auf die zentrale Bedeutung von Übergewicht und Achsfehlstellungen als prädominante Faktoren in der Arthroseentstehung gegenüber Sport hingewiesen werden, die sich in ihrer Kombination potenzieren [33] und deutlich höher als die Sportpartizipation einzuschätzen sind.

Die Fortführung von Bewegungstherapie und Sport bei bereits eingetretener Arthrose erscheint mit geringen Einschränkungen der Gelenkfunktion verbunden, und lässt bei entsprechender Ausführung gute sekundärpräventive Effekte erwarten. Darüber hinaus ist der allgemeine präventive Effekt von Ausdauersportarten – wie Laufen – auch sportmedizinisch abgesichert. Neuere Studien konnten zeigen, dass moderates Training, vor allem mit isometrischen und isokinetischen Trainingsmethoden, die klinische Performance von Arthrosepatienten deutlich verbessern konnte, auch bewegungsarme Patienten profitierten von strukturierten Aktivitätsprogrammen. Problematisch ist einerseits die Implementierung von nachhaltigen Präventionsprogrammen, die über die Jahre durchgeführt werden müssen und andererseits, dass es eine geringe Dosis-Wirkungs-Korrelation gibt, hinsichtlich der Effizienz von Sporttherapie [34, 35].

Die häufigste Komorbidität mit Arthrose ist das Übergewicht, wobei vor allem der Fettanteil entscheidend ist für die Korrelation zur Osteoarthroseinzidenz, daher ist die Modifikation der Ernährungsgewohnheiten nicht nur eine qualitative, sondern auch eine quantitative Maßnahme, um Adipositas abzubauen. Daher ist das Übergewicht der wichtigste modifizierbare Risikofaktor, wobei hier der Grenzwert bei einem BMI von über 27–30 liegen dürfte, der zur massiven Erhöhung der Arthroseinzidenz führt.

Hinsichtlich Alter und Geschlecht sind Frauen fast doppelt so häufig betroffen wie Männer

Wenn wir nun die Faktoren der Arthroseentstehung und der möglichen Beeinflussung gegenüberstellen, können wir folgende Risikofaktoren für Osteoarthrose feststellen: Hinsichtlich Alter und Geschlecht sind Frauen fast doppelt so häufig betroffen wie Männer, wobei es in beiden Altersgruppen mit zunehmendem Alter zur Erhöhung und Angleichung der Inzidenz von Arthroseveränderungen kommt. Die Arthroseprozesse sind auch von der hormonellen Situation beeinflusst, wobei die Arthroseentstehung mit Veränderung von Geschlechtshormonrezeptoren im Knorpel einhergeht. Der genetische Hintergrund erscheint mehr und mehr von Bedeutung, wobei hier einerseits die angeborenen Gelenkachsen betroffen sind, andererseits aber auch spezifische genetische Veränderungen, speziell im Kollagenstoffwechsel, mit erhöhten Arthroseraten verbunden sein können. Angeborene muskuloskelettale Fehlanlagen, anatomische Variationen und Dysplasien stellen einen weiteren Block schwer zu beeinflussender, prädisponierender Faktoren dar und lassen den Begriff der idiopathischen Arthrose etwas relativ erscheinen, da keine optimale anatomische Gelenkanatomie vorgegeben ist. Zuletzt müssen neben Stoffwechselerkrankungen mit Gelenkbeteiligung, wie der Gicht, auch Erkrankungen mit rheumatischem und immunologischem Hintergrund in die Betrachtung eingeschlossen werden.

Die Gesamtbelastung ist treibender Faktor für die Beurteilung des Arthroserisikos

Die physische Aktivität und Bewegungsinzidenz sind auch entscheidend mit der Ausbreitung von schmerzhaften Veränderungen des Bewegungsapparates verknüpft. Zu beachten bleibt, dass der Arthrosegrad oft nicht mit dem Ausmaß der Schmerzsymptomatik korreliert. Koordinative Defizite und mangelnde muskuläre Sicherung im Bereich der Band-Gelenk-Kapsel und Sehnenansätze führen zu schmerzhaften Veränderungen, die mit Arthrose kombiniert sein können. Oft ist die Klinik des Patienten vordergründig darauf zurückzuführen und weniger durch den Knorpelschaden selbst, daher ist eine differenzierte klinische Untersuchung wichtig. Irreführend kann die isolierte Betrachtung von Sport sein, ohne die Belastungen aus Freizeit und Arbeit miteinzuberechnen. Denn die Kumulation davon, also die Gesamtbelastung, ist treibender Faktor für die Beurteilung des Arthroserisikos.

Trotzdem erscheint die Erhaltung der Bewegung in der Arthrosedynamik wichtig und es wurden daher in der Publikation von Roddy und Zhang [34] diesbezügliche evidenzbasierte Empfehlungen für das Management der Osteoarthrose der Hüfte und des Knies im Move-Konsensus publiziert. Hier wurde festgestellt, dass die Evidenz im Zusammenhang mit Sport und Arthrose einen generell positiven Effekt gibt, und dass diese spezifischen Sportempfehlungen zwar wirksam sind, aber es nur zu einer geringen Dosis-Wirkungs-Korrelation kommt. Insgesamt wird das Widerstandstraining im Sinne eines isometrischen Trainings empfohlen. Vordergründige Rationale für die Sportempfehlung bei Arthrose ist die Wiederherstellung der muskulären Kraft und Koordination. Somit kann und soll das oben beschriebene Zusammenspiel von Immobilität und Arthroseprogression unterbrochen werden. Diese Verbesserung der Gelenksfunktion führt zur Linderung des Reizzustandes und damit zu einer Schmerzreduktion, die sich auch in der Knorpelmorphologie mit einem Erhalt der Knorpeldicke verifizieren lässt.

Die Sporttherapie muss in Bewegungsprogrammen durchgeführt werden [35], die zunächst physiotherapeutisch geübt und gelernt werden sollen. Dringend zu empfehlen ist dabei die Erhebung des Ausgangszustandes, um eine individuelle Abstimmung auf Bewegungsumfang und Intensität zu gewährleisten und Überbelastung zu vermeiden. Voraussetzung ist hier auch, wie überall im Sport, eine sportmedizinische Untersuchung, um die generelle Sporttauglichkeit festzustellen. Letztendlich müssen die Bewegungsprogramme auch im Rahmen eines Heimtrainings durchgeführt werden. Es hat sich gezeigt, dass der Erfolg solcher Programme nur durch fix geplante Nachuntersuchungen und Auffrischungseinheiten erreicht werden kann [36, 37].

## Sportberatung bei Arthrose und Sporttauglichkeit

Die Beratung bei Arthrose zielt vor allem auf die Erhebung der anamnestischen Vorschädigung und Erkrankungen ab. Eine genaue Anamnese von Traumata und deren nachfolgender Behandlung mit der Erfragung der Dauer von Ruhigstellungen ist wichtig. Aber auch Erkrankungen wie Hüftdysplasien, Bindegewebs- und Stoffwechselerkrankungen müssen erfasst werden.

Nachfolgend ist eine Erhebung der Gelenkachsen sowie Gelenk- und Muskelfunktionen wichtig, um die Funktionalität des muskuloskelettalen Systems zu dokumentieren. Schmerzhafte Sensationen sind diagnostisch zu erfassen, wobei auch eine radiologische Abklärung anzuraten ist. Bei persistierenden oder inadäquaten rasch zunehmenden Schmerzsensationen sollte auch eine MRT zur Abklärung der Gelenkstrukturen, vor allem im Hinblick auf Knochenmarködeme, Synovitis mit Infekt und Tumor, durchgeführt werden. Die muskuläre Stabilisierung und die damit verbundenen koordinativen Fähigkeiten können durch einfache Übungen und Tests, wie den Einbeinstand, Up-and-Go oder Gehdistanztests, erhoben werden. Bei der sportspezifischen Anamnese ist vor allem entscheidend, inwieweit die Sporttechnik vor Entstehung der Arthrose schon beherrscht wurde. Beim Neuerlernen von Sportarten mit arthrotischen Gelenken ist oft die mangelnde Koordination ein Hindernis, da die schmerzhafte Blockade oder eingeschränkte Gelenkfunktionalität ein Erlernen der Bewegungen kaum möglich macht.

Besonders geeignet für Arthrosepatienten erscheinen Sportarten wie Radfahren, beziehungsweise Ergometertraining, Wandern mit Stöcken oder Nordic Walking, Skilanglauf im klassischen Stil oder Wassergymnastik [32, 38]. Speziell beim Radfahren erscheint die zyklische Bewegung in entlastender Sitzposition für die untere Extremität besonders günstig. Bei schon eingeschränkter Beweglichkeit ist vor allem das Fahrradergometer zu empfehlen, was die Koordination zum Auf- und Abstieg erleichtert. Durch das Verwenden eines Damenrades oder eines Rades mit niedrigem Holm kann das Absteigen im Gelände gut erleichtert werden. Eine entsprechende Gangschaltung, aber auch die Wahl der Touren und die Ausrüstung sind hier entscheidend für die Gelenkbelastung. Beim Nordic Walking ist die Verminderung der Belastung im Bereich der Gelenke der unteren Extremität doch etwas geringer, als zunächst angenommen. Durch die Verwendung der Stöcke wird der Schritt etwas größer, somit auch teilweise die Krafteinwirkung höher, sodass dies nur bei gutem Beherrschen der Sportart empfohlen werden kann. Uneingeschränkt sind aber Gehstöcke beim Bergwandern zu empfehlen, diese sollten aber längenverstellbar sein und variable Griffhöhen haben, damit die Abstützung optimal ans Gelände angepasst werden kann.

Sportarten mit höherem technischem Anspruch sollten – wie bereits erwähnt – schon vor der Arthroseentstehung beherrscht werden, da ein Erlernen mit Arthrose schwierig oder unmöglich ist. Zu diesen Sportarten zählen vor allem Tennis, Golf, Skilauf, Tischtennis, Segeln und Reiten. Bei entsprechender Erfahrung und Akzeptanz des etwas eingeschränkte Leistungsniveaus, können diese Sportarten durchaus weiter betrieben werden. Wichtig erscheint hier das Verwenden von gedämpften Schuhen, eventuell Gehhilfen, Orthesen oder beim Golfen das Verwenden des Carts. Die Modifikationen der Technik – wie beim Tennis ohne ausgeprägte Rumpfrotation oder beim Golfen unter Durchführen des Golfschwunges ohne entsprechender Körperverdrehungen und Knieausgleichsbewegung – sind diese Sportarten durchaus sinnvoll. Ungeeignete Sportarten bei Arthrose sind Mannschaftssportarten oder Sportarten, die mit hohem Tempo und nicht vorhersehbarem Richtungswechsel oder Gegnereinwirkung verbunden sind. Hier sind zum Beispiel Squash, Trampolinspringen, Basketball, Handball, Fußball oder auch Disziplinen wie Gewichtheben und leichtathletische Disziplinen anzuführen.

## Gelenkentlastung als Arthroseprävention

Als Prinzip der Arthroseprävention können zyklische Belastungen und Bewegungen als zentraler Faktor genannt werden. Zyklische Belastungen erhalten die Funktionalität von Knorpel und Gelenk insbesondere durch die dadurch verbesserte Ernährung der Knorpelzellen durch Diffusion. Um diesen Effekt optimal nutzen zu können, müssen diese Bewegungen allerdings bei möglichst minimierter Belastung und Krafteinwirkung ausgeführt werden. Daher stellt Radfahren oder Wandern mit Stöcken eine sehr geeignete präventive Intervention dar. Weiter ist die Anwendung von entlastenden Orthesen bei den frühen Arthrosen sicher sinnvoll. Hier können knieübergreifende oder korrigierende Fuß- und Unterschenkelorthesen helfen, den Knorpel und den subchondralen Knochen zu entlasten. Die Fußunterschenkelorthesen (z. B. Agilium Freestep, Otto Bock HealthCare, Duderstadt, Deutschland) haben den Vorteil, dass sie zu keiner Muskelatrophie führen und die muskuläre Gelenkstabilität erhalten bleibt. In einer vergleichenden Studie konnte gezeigt werden, dass die Schmerzsituation durch beide entlastenden Orthesen deutlich verbessert werden kann. Ähnliches gilt auch für die Verwendung von Einlagen mit geringer lateraler Keilung bei beginnenden medialen Arthrosen, was ebenfalls die mechanische Gelenkachse aus dem überlasteten medialen Kompartiment bringt. Die dadurch gesetzten Maßnahmen können verhindern, dass Reizzustände entstehen, damit der entzündliche Zustand, der den Knorpel nachhaltig schädigen kann, verhindert wird.

## Behandlung der frühen Arthrose

Tritt im Zuge einer Bewegungsbelastung oder oft auch ohne Anlass ein Reizzustand im Sinne einer Aktivierung der Arthrose auf, ist sofort ein passageres Sportverbot einzuhalten, bis die Schmerzen, der Erguss und die Überwärmung des Gelenkes abgeklungen sind. Kältetherapien lokal und generell, passagere komplette Entlastung mit Krücken und abschwellende physikalische Maßnahmen sowie eine lokale oder/und systemische antiinflammatorische Therapie ist einzuleiten [39]. Grundsätzlich unterscheiden wir zwischen der topischen, systemischen und infiltrativen Therapien.

Die lokal topische Behandlung hat sich hier als sehr gut verträglich etabliert, wobei NSAR-Gele auch zusammen mit Ultraschallbehandlungen angewandt werden. Im Idealfall reduziert die Behandlung sowohl die Schmerzen als auch das Fortschreiten der Arthrose, was bislang noch für kein Medikament ausreichend bewiesen ist. Durch die topische Behandlung mit NSAR kann bei 60 % der Patienten eine 50 %ige Schmerzreduktion erreicht werden [40]. Paracetamol wird häufig für Arthroseschmerzen verwendet, wenngleich die Wirksamkeit hier unter den Entzündungshemmern liegt. Capsaicin kann bei moderater Arthrose als lokale Schmerztherapie eingesetzt werden. Die Infiltration von Hyaluronsäure wird international kontrovers diskutiert, da die Studien keinen minimalen relevanten Therapieeffekt nachweisen können, was dem hohen Placeboeffekt der Kontrollgruppen geschuldet ist. In experimentellen Studien konnte ein antientzündlicher Effekt nachgewiesen werden, sowie direkte Effekte des Hyaluronsäure-Rezeptors CD44 anzunehmen sind [41]. Durch das hohe Molekulargewicht der therapeutisch eingesetzten Hyaluronsäure wird anstatt einer Aktivierung die gewünschte Hemmung von CD44 erreicht. Die verschiedenen Formen von „platelet rich plasma“ (PRP) besitzt ebenfalls antientzündliche Eigenschaften und werden vor allem in der leukozytenarmen Form eingesetzt. In einem systematischen Review wurde ein positiver Effekt von PRP bei Arthrose nach 12 Monaten konstatiert [42].

Die Infiltration von Glukokortikoiden wird aufgrund der Wirksamkeit bei Gelenksergüssen bzw. Begleitsynovitiden, die in ca. 50 % der Fälle auftreten, häufig praktiziert, sollte aber nicht mehr als dreimal pro Jahr wiederholt werden. Die intraartikuläre Injektion von Steroiden führt zur Veränderungen der Zellaktivität der Knorpelzellen im Sinne eines katabole Zustandes, der zu einer Verminderung des Knorpelvolumens und einer Apoptoseinduktion führen kann [43]. Der oft gleichzeitig durchgeführten intraartikulären Applikation von Lokalanästhetika wird auch eine Knorpeltoxizität zugeschrieben, was aber oft in Kombination angewendet wird. Beide Effekte können durch die Beigabe von Hyaluronat gehemmt und verbessert werden, sodass jetzt zunehmend Mischpräparate mit Hyaluronat und Kortison angeboten werden.

Eine zuletzt publizierte Studie von Filardo mit vergleichender Metaanalyse der injizierbaren Knorpeltherapeutika zeigte vor allem bei längeren Beobachtungszeiträumen Vorteile von PRP gegenüber Hyaluronsäure und insbesondere gegenüber Cortison, das nur sehr kurz wirksam ist und darüber hinaus ausgeprägte Veränderungen in der Gelenkhomöostase bedingt [44].

Die Unterstützung mit „Chondroprotektiva“ kann sinnvoll sein, sollte aber nur als flankierende Maßnahme gesehen werden. Hier kann keine definitive Entscheidung bezüglich der Substanz, in welcher Form und Dosis hier behandelt werden soll, gegeben werden. Im klinischen Alltag wird Chondroitin und Glukosamin eher in der Frühphase der Arthrose angewendet, wenn die Syntheseleistung der Knorpelzellen noch intakt und der Knorpel-Matrix-Aufbau noch als realistisch einzuschätzen ist, was aber prinzipiell als sehr eingeschränkt angesehen wird. Chondroitin und Glukosamin sind weitgehend ohne Nebenwirkungen, haben aber auch ein geringes antiinflammatorisches Potenzial und verfügen – wie auch andere Nahrungsergänzungsmittel – über einen starken Placeboeffekt. Es liegen positive Studien zu Glukosamin (1500 mg/Tag) oder Chondroitin (800 mg/Tag) vor, allerdings ist deren Effekt insgesamt als gering einzuschätzen [45]. In einem systematischen Review wurden 20 verschiedene Nahrungsergänzungsmittel bei Knie‑, Hand- und Hüftarthrose untersucht, wobei klinisch signifikante aber nur kurzfristige Schmerzwirksamkeiten (<3 Monate) für mehrere Substanzen berichtet wurden (L-Carnitin, Pinienrindenextrakt, Weihrauchextrakt, Curcuma-longa-Extrakt, Passionsfruchtextrakt und Kollagenhydrolysat) [46]. Vier weitere Substanzen hatten nur tendenzielle, gering signifikante Wirksamkeit (undenaturiertes Kollagen Typ 2, Avocado, Sojabohnen-Bestandteile, Methylsulfonylmethan und Diacerein).

In einer Studie mit Vitamin D konnte kein Vorteil gegenüber Placebo in Bezug auf Schmerz und Knorpelvolumen gezeigt werden [47], wobei aber die Erhaltung des subchondralen Knochens und die Vermeidung von periartikulärer Osteopenie wichtig erscheint. Daher zielen neuere Behandlungsansätze auf den pathologischen subchondralen Knochenumbau, welcher bei der Arthrose häufig noch vor dem Knorpelschaden auftritt, wie wir dies in den digitalen Analysen des subchondralen Knochens auch gesehen haben. Hier zeigte sich in verschiedenen Studien ein positiver Effekt von Bisphosphonaten und Strontiumranelat [48]. Letzteres wurde allerdings aufgrund einer Sicherheitswarnung nicht weiterverfolgt.

Zuletzt bleibt die Akzeptanz des Patienten gegenüber bestehenden Schäden, was besonders bei frühen arthrotischen Veränderungen die Wahl von Sportarten und Belastungen betrifft, die der eingeschränkten Tragfähigkeit gerecht werden. Diese psychologische Komponente und die persönliche Motivation ist im Therapie- und Betreuungskonzept gleichberechtigt mit den anderen Interventionen umzusetzen. Bei Berücksichtigung der beschriebenen Faktoren kann durchaus auch beim Vorliegen arthrotischer Veränderungen noch befriedigend Bewegung und Sport durchgeführt werden, was mit dem Erhalt der Mobilität und der eigenen Gelenke im Altersgang ein wesentlicher Beitrag zur Lebensqualität ist.

## Fazit für die Praxis

- Osteoarthrose ist ein weitverbreitetes Krankheitsbild welches zu einer zunehmenden Beeinträchtigung der Mobilität von Betroffenen führt. Im Zentrum der Genese steht die Trias aus Alter – Degeneration – Inflammation.
- Aufgrund des demografischen Wandels wird die relative und absolute Zahl Betroffener zunehmen.
- Prävention spielt eine entscheidende Rolle. Die Früherkennung hilft, präventive Maßnahmen rechtzeitig zu setzten.
- Zukünftig können Methoden der künstlichen Intelligenz mithelfen, objektiver und frühzeitiger zu diagnostizieren und zu therapieren.
- Die konservative Therapie kennt viele Methoden, die auch „regenerative“ Therapien wie autologe Blutprodukte in einen evidenzbasierten Behandlungsalgorithmus einfließen lassen. Sport- und Bewegungsadaptation steht dabei an oberster Stelle.
- Ziel der konservativen Therapie ist es, durch die Kombination mehrerer Methoden – Lebensstiladaptation, Sportanpassung, Gelenkshomöostase, muskuläre Balance etc. – die Mobilität zu erhalten, zu fördern und eventuell nötige Operationen hinauszuzögern oder obsolet werden zu lassen.

## Abkürzungen

CD: „Cluster of differentiation“
IL: Interleukin
NSAR: Nichtsteroidale Antirheumatika
PRP: „Platelet rich plasma“
TGF: „Transforming growth factor“
TNF: „Tumor necrosis factor“
